# LGMMFusion: A LiDAR-guided multi-modal fusion framework for enhanced 3D object detection

**DOI:** 10.1371/journal.pone.0331195

**Published:** 2025-09-04

**Authors:** Haixing Cheng, Chengyong Liu, Wenzhe Gu, Yuyi Wu, Mengye Zhao, Wentao Liu, Naibang Wang

**Affiliations:** 1 China Coal Energy Research Institute Co., Ltd., Xi’an, Shaanxi Province, China; 2 School of Mechanical and Electrical Engineering, China University of Mining and Technology (Beijing), Beijing, China; Universidade Federal de Uberlandia, BRAZIL

## Abstract

Multi-modal data fusion plays a critical role in enhancing the accuracy and robustness of perception systems for autonomous driving, especially for the detection of small objects. However, small object detection remains particularly challenging due to sparse LiDAR points and low-resolution image features, which often lead to missed or imprecise detections. Currently, many methods process LiDAR point clouds and visible-light camera images separately, and then fuse them in the detection head. However, these approaches often fail to fully exploit the advantages of multi-modal sensors and overlook the potential for enhancing the correlation between modalities before feature fusion. To address this, we propose a novel LiDAR-guided multi-modal fusion framework for object detection, called LGMMfusion. This framework leverages the depth information from LiDAR to guide the generation of image Bird’s Eye View (BEV) features. Specifically, LGMMfusion promotes spatial interaction between point clouds and pixels before the fusion of LiDAR BEV and image BEV features, enabling the generation of higher-quality image BEV features. To better align image and LiDAR features, we incorporate a multi-head multi-scale self-attention mechanism and a multi-head adaptive cross-attention mechanism, using the prior depth information from point clouds to generate image BEV features that better match the spatial positions of LiDAR BEV features. Finally, the LiDAR BEV features and image BEV features are fused to provide enhanced features for the detection head. Experimental results show that LGMMfusion achieves 71.1% NDS and 67.3% mAP on the nuScenes validation set, while also improving the detection of small objects and enhancing the detection accuracy of most objects.

## Introduction

With the rapid development of autonomous driving technology, environmental perception systems play a crucial role in ensuring the safety and efficiency of autonomous driving [1–6]. Among various perception challenges, small object detection is particularly critical, as pedestrians, cyclists, and small obstacles often suffer from sparse LiDAR points and low-resolution image features, leading to missed detections or inaccurate localization. The failure to accurately detect small objects in time can result in severe safety risks, especially in complex urban environments with dense traffic and dynamic obstacles [7]. As one of the core technologies of perception systems, 3D object detection provides high-precision awareness of the surrounding environment, directly influencing the decision-making process of autonomous driving systems [8–11]. Accurately identifying and understanding various objects on the road, including small and distant objects, is fundamental to ensuring safe and rapid responses in autonomous driving scenarios [12]. As autonomous driving applications expand to increasingly complex environments, improving the accuracy and robustness of 3D object detection algorithms remains one of the key technical challenges [13].

Although sensor fusion technology has made significant progress, particularly in the field of 2D object detection, the application of 2D detection algorithms in autonomous driving faces many challenges [14,15]. Traditional 2D object detection methods, such as Faster R-CNN [16] and YOLO [17], are capable of handling object recognition tasks in images, but due to the lack of depth information, they struggle to accurately determine the spatial position and orientation of objects. 2D detection methods typically treat images as flat, ignoring the spatial relationships of objects in three-dimensional space, which leads to poor detection performance, especially for distant or small objects, in complex 3D scenarios [18]. As the demand for perception accuracy and adaptability to complex environments in autonomous driving increases, the limitations of 2D detection methods become more apparent. Therefore, 3D object detection, as a core technology to address this issue, has become increasingly important [12].

In autonomous driving perception systems, commonly used sensors include LiDAR, radar, cameras, GPS, and inertial measurement units (IMU) [5,8]. Each sensor has its unique advantages and limitations: LiDAR provides high-precision 3D point cloud data but is limited by occlusion and cost in complex environments [19]; radar performs well in harsh weather conditions and is suitable for long-range detection, but it has lower resolution [20]; cameras provide rich visual information, capable of recognizing traffic signs and pedestrians, but perform poorly under low light and adverse weather conditions and lack depth information. To compensate for the limitations of each sensor, effectively fusing data from multiple sensors to create a high-quality 3D environmental model has become a focal point in current autonomous driving research [21].

As mentioned earlier, a single data source is insufficient to meet the comprehensive perception needs of autonomous driving systems. By fusing information from different sensors, the advantages of each sensor can be fully utilized, overcoming the limitations of individual sensors and improving perception capabilities in complex environments. Traditional multimodal fusion methods are generally categorized into early fusion, late fusion, and intermediate fusion [22,23]. Early fusion directly combines image information with point cloud data, which helps with local object localization but has limitations in data alignment and information preservation. Late fusion merges 2D and 3D detection boxes after extraction, though simple and effective, it may not fully leverage the complementarity of the data sources.

However, most existing fusion methods struggle with small object detection due to their inability to effectively combine fine-grained spatial and contextual information from multiple modalities. Small objects often contain fewer LiDAR points, making it difficult for late-fusion approaches to improve detection accuracy significantly. Similarly, early-fusion methods may suffer from misalignment issues that further degrade performance. Thus, achieving more refined feature fusion and establishing more accurate spatial relationships between different sensors has become a key issue in current research [24–26].

Among existing multimodal fusion methods, intermediate fusion techniques are gradually emerging [27,28]. Intermediate fusion methods integrate image and point cloud data at the intermediate stage of feature extraction, which not only avoids the information loss encountered in early fusion but also allows for more efficient data alignment and feature integration through deep learning techniques [29,30]. For example, F-PointNet [31] reduces the search area by introducing 2D detection boxes into the 3D space, while PointPainting [32] enhances detection performance by directly attaching image semantic information to point cloud data. In recent years, many researchers have proposed intermediate fusion-based algorithms, such as BEVFusion [33] and DeepFusion [34], which have achieved significant results by fusing image and point cloud features in the BEV space.

However, despite these advancements, most existing methods still struggle with small object detection, as they do not explicitly enhance the interaction between LiDAR and image features before fusion. This limitation motivates the need for a novel multimodal fusion framework that fully utilizes the complementary characteristics of different modalities while preserving fine-grained details [35] [37].

In this study, we introduce LGMMfusion, a novel framework for multimodal object detection that leverages intermediate fusion strategies. Unlike conventional approaches that focus on late fusion or early fusion, LGMMfusion combines LiDAR and camera data through a deep integration of features in the BEV space, employing spatial queries and self-attention mechanisms. This design enables more accurate spatial alignment of the two modalities, enhancing the robustness of the object detection task. By directly generating BEV features from the image in the BEV space, LGMMfusion establishes more precise correspondences between LiDAR and camera data, minimizing the need for complex data alignment. Our experimental evaluations, conducted on the nuScenes dataset[38], demonstrate that LGMMfusion outperforms traditional LiDAR-only methods, achieving particularly notable improvements in the detection of small objects. The ability to combine multi-scale spatial features from both modalities allows LGMMfusion to effectively utilize complementary information, delivering superior performance in complex environments.

This research holds significant theoretical and practical value [39]. Theoretically, LGMMfusion contributes to the advancement of multimodal fusion methodologies by introducing novel feature alignment and adaptive fusion mechanisms, which can inspire future research in 3D perception. Practically, the framework enhances the reliability of autonomous driving perception, particularly in complex urban environments where detecting small objects is crucial for safety. By improving detection accuracy, especially for pedestrians and distant obstacles, our approach has the potential to reduce traffic accidents and contribute to the broader adoption of autonomous driving technology.

The main contributions of this study are as follows:

- We propose the LGMMfusion framework, which efficiently integrates LiDAR and camera features in the BEV space. We introduce multi-head multi-scale self-attention and multi-head adaptive cross-attention mechanisms, which fully exploit the complementary strengths of LiDAR and camera data to generate high-quality BEV features.
- We design a novel fusion module that employs an adaptive cascading approach. This method effectively balances the fusion weights of LiDAR and camera data, simplifying the multimodal fusion process and improving the accuracy of 3D object detection.
- Extensive experiments on the nuScenes dataset demonstrate that LGMMfusion outperforms traditional LiDAR-only methods. It achieves significant performance gains across a wide range of object sizes and environmental conditions, achieving 71.1 NDS and 67.3 mAP, and demonstrating particularly strong detection capabilities for small objects.

## Related work

In this section, we review the key approaches in 3D detection, focusing on LiDAR-only methods, camera-based techniques, and multi-modal fusion strategies. These methods provide diverse perspectives and solutions, each contributing to advancements in 3D object detection.

### LiDAR-based 3D detection

LiDAR-based 3D object detection is crucial in autonomous driving. Various methods have been proposed to process LiDAR point cloud data for accurate object detection and localization. Early research focused on point-based methods, such as PointNet [36,40] and SparseConvNet [41], which directly process raw point clouds. While these methods handle irregular point cloud data well, they are relatively slow and computationally inefficient. To improve speed and efficiency, voxel-based methods emerged, discretizing point clouds into regular 3D grids for efficient sparse convolutions. Notable works, such as VoxelNet [42] and SECOND [43], significantly enhanced computational efficiency by employing sparse convolutions and generating prediction results in the BEV space.

In addition to point-based and voxel-based approaches, range view representation has also gained attention in LiDAR 3D object detection [44]. This representation projects point clouds into an image-like space, simplifying computations. However, it often loses geometric detail, leading to suboptimal performance compared to other methods. Recent research has also introduced anchor-free designs, which eliminate the need for traditional anchor boxes, enhancing object representation and flexibility. Furthermore, Transformer architectures have been applied to LiDAR-based 3D detection [45,46], particularly for feature extraction and object representation, showing promising results despite higher computational complexity.

### Camera-based 3D detection

Due to the high cost of LiDAR sensors, camera-based 3D perception has received significant attention. Camera-based 3D object detection methods typically operate using single-view or multi-view images. In the case of single-view images, some approaches attempt to predict 3D bounding boxes directly from image features or leverage intermediate representations for object detection [47,48]. For multi-view inputs, image features are optimized within a constructed 3D geometry volume, or projected and merged into the frustum space with predicted depth. However, since the accuracy of predicted depth maps is inferior to LiDAR, this leads to semantic ambiguity in the BEV space. To address this issue, many methods use geometric cues, such as extracting geometric information from multi-view images in an implicit manner, though this approach sacrifices direct spatial interactions.

Recent studies have introduced view transformation methods, using view transformers to convert camera features from perspective to BEV space [49,50]. These methods, inspired by LiDAR-based designs, aim to enhance the accuracy of 3D detection by transforming from perspective to BEV. For instance, BEVDet [51] and M2BEV [52] extend the LSS and OFT frameworks, incorporating explicit supervision for depth estimation. However, the accuracy of depth estimation remains a challenge for purely camera-based detection. Some methods, such as pseudo-LiDAR techniques, have been proposed to mitigate this limitation by converting RGB images into point clouds using pre-trained depth estimation networks [56]. These point clouds can then be integrated with standard LiDAR detectors. Another approach involves converting camera features into BEV representations, such as BEVFormer [49] and Bevformerv2 [50], which use spatiotemporal cross-attention to compute feature interactions across frames, enabling more efficient 3D object detection.

In summary, while camera-based methods have made significant progress in 3D object detection, they still face challenges with depth estimation and semantic ambiguity. The integration of geometric cues and the use of view transformers show promise in improving performance, but challenges remain when compared to LiDAR-based methods. The fusion of camera and LiDAR data is often seen as an effective way to address these limitations.

### Multi-modal fusion for 3D detection

With the development of multi-sensor fusion technology, multi-modal fusion for 3D object detection has become a research hotspot [57–59]. By combining data from different sensors, such as LiDAR and cameras, the strengths of each sensor can be leveraged to enhance 3D object detection performance. These methods can be classified into proposal-level fusion, point-level fusion, and intermediate-level fusion. Each of these fusion strategies presents distinct trade-offs. Proposal-level fusion supports object-centric reasoning and efficient feature extraction but often depends on accurate region proposals and suffers from limited spatial alignment precision. Point-level fusion provides detailed semantic augmentation by projecting image features onto point clouds; however, it is susceptible to projection noise and lacks global context integration. Intermediate-level fusion strikes a balance by performing feature interaction in the BEV or semantic space, enabling richer cross-modal understanding while preserving structural consistency. Moreover, recent research efforts have emphasized the importance of flexible and context-aware fusion mechanisms across multiple domains. For instance, Wang et al. [60] proposed a spatio-temporal deep learning model for streamflow prediction based on multi-source data fusion, highlighting the benefit of integrating heterogeneous data sources. Similarly, Wang et al. [61] developed a hybrid Autoformer-ELM framework to enhance multi-step carbon price forecasting using diverse influencing factors. These studies, although focused on environmental prediction tasks, reinforce the value of adaptive and dynamic fusion architectures — a principle that underlies our design of LGMMfusion’s deformable alignment and weighted fusion strategy.

Proposal-level fusion methods were first introduced in MV3D [62], which generates object proposals in 3D space and projects them onto images to extract Region of Interest features. Several improvements have since been proposed, such as Frustum-PointNet [31] and Frustum-ConvNet [63], which elevate image proposals to 3D frustums to identify relevant regions in point clouds. FUTR3D [64] and TransFusion [65] generate object queries in 3D space and fuse image features into these proposals. These methods are object-oriented and suitable for object-level tasks, but are difficult to apply directly to other tasks, such as BEV map segmentation. Point-level fusion methods enhance the semantic information of point cloud data by projecting image semantic features onto foreground points of LiDAR data. These methods include PointPainting [32] and PointAugmenting [66], where the former projects 2D semantic segmentation results onto point clouds, and the latter annotates point clouds using deep CNN features. This approach performs well in complex scenarios because it integrates rich semantic information from images directly into the geometric structure of LiDAR point clouds.

Intermediate-level fusion methods have received increasing attention in recent years. For example, ContinuousFusion [67] and DeepFusion [34] perform fusion at the deep feature level, enhancing cross-modal interaction by sharing information between 2D and 3D backbone networks. Xu et al. [53] proposed the FusionPainting framework to fuse 2D RGB images and 3D point clouds at the semantic level, enhancing 3D object detection. However, it did not fully utilize LiDAR depth information. BEVFusion improves the accuracy and robustness of multi-modal fusion by projecting image features onto the BEV space and integrating them with LiDAR data. Unlike BEVFusion, which relies on purely image-based depth estimation to construct BEV representations, LGMMfusion uses LiDAR depth to supervise the transformation, reducing spatial ambiguity. DeepFusion performs late-stage feature blending with limited geometric alignment, whereas our adaptive cross-attention explicitly models point-pixel interactions using deformable offsets. Beyond the field of autonomous driving, recent efforts in environmental modeling have also demonstrated the importance of multi-source data fusion and interpretability in deep learning. For example, Wang et al. [54] proposed a secondary modal decomposition ensemble model for groundwater prediction, while Li et al. [55] developed an interpretable deep learning framework for water quality analysis. These works underscore the cross-domain relevance of integrating heterogeneous data for robust and explainable prediction.

## Proposed fusion method

**Fig 1:**
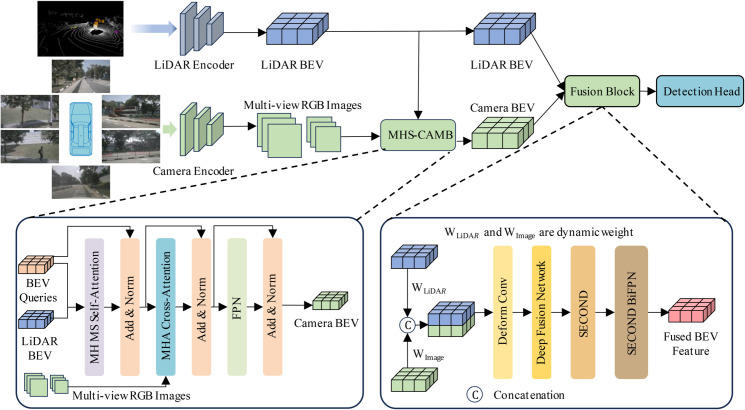
Our LGMMfusion framework initially extracts features from LiDAR point clouds and camera images through their respective backbone networks. Subsequently, the accurate depth information from the LiDAR is harnessed to guide the multi-view images in generating Image BEV representations. Ultimately, these image BEVs are integrated with the LiDAR BEVs to achieve a comprehensive fusion.

In this section, we introduce the LGMMfusion framework, a novel approach that effectively integrates multi-modal features from LiDAR and camera data to enhance 3D object detection tasks. The LGMMfusion framework is designed to perform deep fusion feature extraction from a BEV perspective, enabling the effective combination of complementary information provided by LiDAR and camera sensors. The framework consists of four main modules, each playing a crucial role in the overall processing pipeline: the feature extraction module, the multi-head self-attention and cross-attention mechanism block, the feature fusion module, and the object detection head, as illustrated in Fig 1. These modules are sequentially organized, where each module progressively builds upon the output of the previous one, creating a unified and effective system for 3D object detection.

Compared to previous intermediate fusion frameworks such as BEVFusion and DeepFusion, LGMMfusion introduces three core innovations: (1) a LiDAR-guided mechanism that enhances the generation of image BEV features through geometric depth priors, (2) an adaptive multi-head cross-attention module that promotes spatially aligned multimodal feature interaction, and (3) a dynamic weighted fusion module that adaptively balances the contributions of LiDAR and image features in a location-aware manner. These innovations collectively improve both the spatial alignment and the discriminative quality of the fused BEV representation, leading to more accurate and robust 3D object detection, especially for small and occluded objects. Each module is described in detail below.

The feature extraction module forms the foundation of the LGMMfusion framework, tasked with capturing essential features from both LiDAR and camera data. This module is responsible for transforming raw sensor inputs into a more informative and structured representation that can be utilized in subsequent stages. Once the features are extracted, the multi-head self-attention and cross-attention mechanism block is employed to further process these features. The self-attention mechanism allows for the identification of spatial dependencies within each modality, while the cross-attention mechanism facilitates the interaction between the multi-modal features, ensuring that the complementary information from both sensors is effectively aligned. The output of this block is then passed to the feature fusion module, where the extracted features are combined into a unified representation. This fusion process not only enhances the depth and richness of the feature set but also promotes more robust object detection by incorporating the strengths of both LiDAR and camera modalities. Finally, the object detection head takes the fused features and applies them to identify and localize objects in the 3D space. This module generates the final detection results, marking the culmination of the feature processing pipeline.

### Feature extraction

For each input modality, we employ respective encoder networks to generate the corresponding feature maps. This section describes the feature extraction process for both raw LiDAR and image data, which are the foundation for subsequent processing in our framework.

#### LiDAR feature extraction.

For LiDAR point cloud data, we begin by converting the raw point cloud into a 3D voxel grid through voxelization. This step discretizes the continuous point cloud into a regular grid, making it suitable for processing by convolutional networks. To extract spatial features from the voxel grid, we utilize a Sparse Convolutional Network, which efficiently handles the sparsity of LiDAR data. The sparse convolutional layers focus on the non-empty voxels, enabling the network to capture essential structural information while maintaining computational efficiency. After passing through the encoder, the BEV feature map for the LiDAR data is denoted as $B_{\text{LiDAR}}\in {\mathbb{R}}^{X\times Y\times D}$, where *X* and *Y* represent the width and height of the BEV perspective, respectively, and *D* denotes the depth or the number of channels in the feature map. The LiDAR encoder contains four 3D sparse convolutional layers with a kernel size of 3×3×3 at each stage. These layers progressively reduce the spatial resolution while extracting high-level geometric features from the voxelized point cloud.

The mathematical representation is as follows:

$$B_{\text{LiDAR}}={\text{Encoder}}_{\text{LiDAR}}(P_{\text{LiDAR}})$$ (1)

where $P_{\text{LiDAR}}\in {\mathbb{R}}^{N\times D}$ represents the raw LiDAR point cloud data, with *N* being the number of points in the cloud. The LiDAR encoder, ${\text{Encoder}}_{\text{LiDAR}}$, processes the input data to yield the BEV feature map $B_{\text{LiDAR}}$, which captures essential 3D spatial features for downstream tasks.

#### Image feature extraction.

For image data, we leverage the Swin Transformer to extract high-level visual features. The Swin Transformer, a hierarchical vision transformer, is particularly effective in capturing long-range dependencies within the image, which is crucial for understanding complex scenes. The image encoder performs feature extraction through a series of convolutional layers, followed by further processing via a Feature Pyramid Network (FPN). The FPN enhances the ability to capture multi-scale image features by constructing a pyramidal representation, where each level corresponds to a different scale of the image. This multi-scale feature extraction enables the model to better handle objects of varying sizes and locations within the scene. The feature map at each scale is represented as $I_{l}\in {\mathbb{R}}^{H_{l}\times W_{l}\times C}$, where *H*_*l*_ and *W*_*l*_ denote the height and width of the feature map at the *l*-th scale, and *C* is the number of channels, which corresponds to the dimensionality of the feature vector at that scale.

The corresponding equation is as follows:

$$I_{l}={\text{Encoder}}_{\text{Image}}(I)$$ (2)

where $I\in {\mathbb{R}}^{H\times W\times 3}$ is the input raw image, with *H* and *W* being the height and width of the image, respectively. The image encoder, ${\text{Encoder}}_{\text{Image}}$, processes the raw image to produce the multi-scale feature map *I*_*l*_, which captures visual information at different spatial resolutions. These multi-scale features are essential for detecting objects at various scales and locations in the image.

The integration of LiDAR and image feature extraction modules ensures that complementary spatial and visual information from both modalities is captured effectively. The subsequent fusion of these features allows the model to leverage the strengths of both sensors, enhancing the overall performance of 3D object detection.

### Multi-head self-cross attention mechanism block

In this section, we introduce the Multi-Head Self & Cross Attention Mechanism Block(MHS-CAMB), which plays a pivotal role in generating high-quality BEV features from both LiDAR and image data. The primary goal of this block is to fuse LiDAR and image features in a multi-scale and attention-driven manner, ensuring the preservation of essential information. The key components of this module are the Multi-Head Multi-Scale Self-Attention Mechanism (MHMS-SA), Multi-Head Adaptive Cross-Attention Mechanism (MHA-CA), and the FPN for hierarchical feature refinement. Additionally, we employ additional addition and normalization layers to ensure proper alignment and feature normalization.

#### Multi-head multi-scale self-attention.

**Fig 2:**
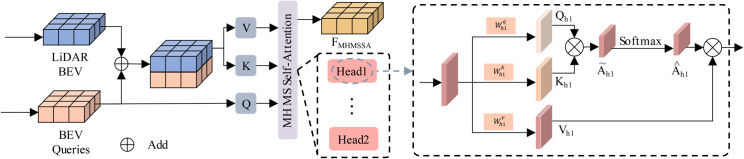
Multi-head multi-scale self-cross attention mechanism block.

The LiDAR BEV features $B_{\text{LiDAR}}$ and BEV queries are processed using the MHMS-SA, as shown in Fig 2. This mechanism is designed to capture features across multiple scales by utilizing deformable attention, which ensures that both high- and low-level features are preserved. While standard Transformer self-attention computes dense pairwise interactions within a flat token sequence, our MHMS-SA module extends this idea to BEV-space feature maps by leveraging multiscale deformable attention. It samples spatially sparse but semantically rich features from both the BEV query and LiDAR feature map across multiple resolutions. This enables MHMS-SA to efficiently aggregate fine-grained spatial details and contextual cues from large receptive fields. Such a design contrasts with traditional Transformer architectures, which require either downsampling or linear projection to approximate multiscale context, often at the cost of spatial precision. By directly attending to multi-resolution feature spaces, MHMS-SA improves detection of objects with varying sizes and spatial sparsity — a key advantage in autonomous driving perception.

For embedded point features, we have the following.

$${𝒜}_{\text{MSA}}(Q_{p},\{Q,B_{\text{LiDAR}}\})=\sum_{V\in \{Q,B_{\text{LiDAR}}\}}\text{DeformAttn}(Q_{p},p,V)$$ (3)

where *Q*_*p*_ represents the sampled points in the BEV grid, Q represents the BEV Query, *p* is the positional information of the sampled points, and *V* is the feature set, which can be either the BEV queries or the LiDAR BEV features.

The deformable attention mechanism is expressed as:

$$\text{DeformAttn}(Q_{p},p,V)=\sum_{m=1}^{M}\sum_{k=1}^{K}W_{m}^{⊤}Q_{p}W_{m}q+KW_{m}k+\varphi (p)+\Delta pC$$ (4)

where $W_{m}^{⊤}$ and *W*_*m*_*k* are the weight matrices for the query and key, respectively; φ(p) is the positional embedding; Δp is the position offset; and *C* represents the feature dimension. This deformable attention mechanism dynamically adjusts the focus on different parts of the feature space, ensuring that spatial relationships are effectively captured across various scales.

#### Multi-head adaptive cross-attention.

**Fig 3:**
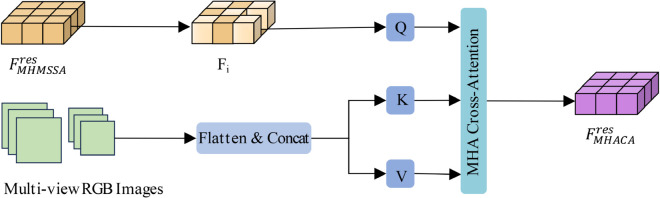
Multi-head adaptive cross-attention block.

**Fig 4:**
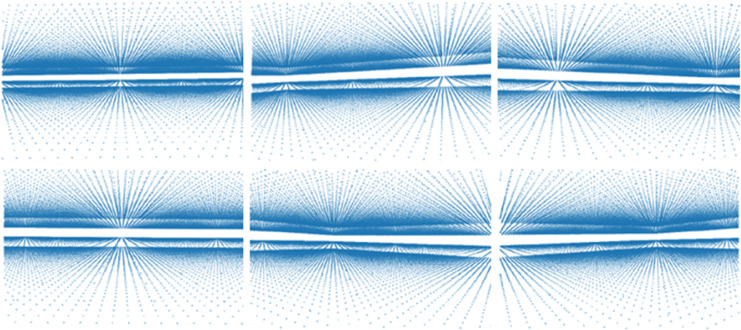
Within the BEV coordinate system, we construct a three-dimensional grid comprising sampling points. The illustrations depict the projections of these 3D grid points onto the 2D image plane as viewed from various angles.

Next, we apply the MHA-CA to align features from the LiDAR and image modalities, as shown in Fig 3. The LiDAR BEV features $F_{\text{IrMesS\_A}}$ guide the generation of BEV features from the image. The cross-attention mechanism ensures that the generated image BEV features are spatially aligned with the LiDAR BEV features, as shown in Fig 4.

The mathematical formulation of this process is:

$${𝒜}_{\text{CreMsCA}}(F_{\text{IrMesS\_A}},\{I_{l}{\}}_{l=1}^{L})=\sum_{i\in V_{\text{hit}}}\sum_{j=1}^{N}\text{MSDeformAttn}(F_{i},p_{ij},\{I_{l}{\}}_{l=1}^{L})$$ (5)

where $V_{\text{hit}}$ denotes the set of reference points associated with specific image perspectives, *p*_*ij*_ is the positional embedding for the reference points, and *F*_*i*_ is the feature processed by the self-attention mechanism.

The MSDeformAttn function computes deformable attention across multiple scales as follows:

$$\text{MSDeformAttn}(F_{i},p_{ij},\{I_{l}{\}}_{l=1}^{L})=\sum_{m=1}^{M}\sum_{k=1}^{K}W_{m}^{⊤}X_{A}mlqk\cdot W_{m}^{\prime }I_{l}\varphi (p_{ij})+\Delta pmlqkC$$ (6)

where *X*_*A*_*mlqk* represents the alignment weights, and $W_{m}^{\prime }$ and *W*_*m*_ are the learned weight matrices for the cross-attention operation. $\varphi (p_{ij})$ is the positional encoding for the reference points, Δp is the position offset, and *C* represents the feature dimension. This cross-attention mechanism ensures effective alignment of LiDAR and image features, maintaining spatial consistency across modalities.

#### Feature pyramid network.

After the multi-head attention operations, the features undergo refinement through a FPN. The FPN generates multi-scale features by progressively refining them through a series of addition and normalization layers. This process helps capture both high-level semantic information and low-level details, making the features more suitable for downstream tasks, such as object detection.

$${ℱ}_{\text{FPN}}=\text{FPN}({𝒜}_{\text{CreMsCA}})$$ (7)

By structuring the attention mechanisms and incorporating key refinements through the FPN, we ensure that the model produces high-quality BEV features that are both spatially coherent and semantically rich. Compared to prior transformer-based fusion methods such as FUTR3D, which rely on global cross-attention to inject image features into 3D object queries, our adaptive cross-attention module introduces several key innovations. First, we use LiDAR-derived geometric priors to guide the sampling positions, improving spatial alignment between modalities. Second, our module applies a multi-head multi-scale deformable attention mechanism, which dynamically selects relevant features from multiple image scales based on BEV query positions. Finally, we incorporate a learnable offset refinement process that adapts sampling points to the underlying scene geometry. These improvements allow LGMMfusion to maintain better consistency in spatial feature correspondence, especially under sparse or occluded input conditions.

### Fusion block

In this section, we introduce the fusion block, which is designed to effectively combine the LiDAR BEV features with the image BEV features to generate the final fused BEV features. This module plays a crucial role in integrating multimodal information, thereby enhancing the performance of multimodal perception tasks. The fusion block consists of the following key steps:

#### Dynamic weighted fusion.

Firstly, the BEV features from LiDAR $F_{\text{LiDAR}}$ and image $F_{\text{Image}}$ are fused using a dynamic weighted mechanism. This fusion process is carried out through a weighted concatenation operation, resulting in the fused feature $F_{\text{concat}}$, which is computed as follows:

$$F_{\text{concat}}=\text{Concat}(W_{\text{LiDAR}}\times F_{\text{LiDAR}},W_{\text{Image}}\times F_{\text{Image}})$$ (8)

Here, $W_{\text{LiDAR}}$ and $W_{\text{Image}}$ are dynamic weight coefficients that control the importance of the LiDAR and image features during the fusion process. These coefficients allow for flexible and adaptive adjustment of the contributions from each modality depending on the context, ensuring that the most relevant features are emphasized in the final fused representation.

#### Deformable convolution and deep fusion network.

After the fusion step, the concatenated feature $F_{\text{concat}}$ undergoes deformable convolution (DeformConv), which adjusts the spatial alignment of the features. Deformable convolutions enable the network to sample features more flexibly in space, allowing for better spatial alignment and improved feature representation, especially in the presence of complex object shapes and spatial variations.

Following the deformable convolution, the fused features are further processed using a Multi-Layer Perceptron (MLP) network for deep fusion. The MLP network captures the non-linear relationships between the features and enhances their representational capacity. Specifically, the fused feature undergoes convolution (Conv) and batch normalization (BN), followed by a ReLU activation function, to obtain the final fused feature:

$$F_{\text{fusion}}=\text{ReLU}(\text{BN}(\text{Conv}(F_{\text{concat}})))$$ (9)

This deep fusion network not only refines the feature representations but also improves the model’s ability to capture complex interactions between LiDAR and image features, thus enhancing the overall perception capability.

#### SECOND algorithm and BiFPN optimization.

The resulting fused feature $F_{\text{fusion}}$ is then passed through the SECOND algorithm for further optimization. The SECOND algorithm utilizes sparse convolutions to improve the efficiency of point cloud processing, optimizing the spatial structure of the fused features. This step allows the model to process large-scale point clouds more efficiently while preserving the important spatial details. The features are then further enhanced through the Bidirectional Feature Pyramid Network (BiFPN), which is capable of fusing multi-scale features more effectively. BiFPN enables the model to focus on features at different spatial resolutions and progressively refine them for more accurate predictions. The final fused feature $F_{\text{second}}$ is computed as follows:

$$F_{\text{second}}=\text{SECOND}(F_{\text{fusion}})$$ (10)

The integration of BiFPN significantly optimizes the fusion process across multiple scales, ensuring that both fine-grained details and broader context are incorporated into the final feature representation. This step not only enhances feature refinement but also contributes to the robustness and accuracy of the multimodal perception system. Through these steps, the fusion block effectively combines the LiDAR BEV features and image BEV features, generating a more accurate and efficient final BEV feature. The dynamic weighted fusion allows for flexible adjustment of feature importance, while deformable convolution and the deep fusion network enhance feature representation. Finally, the SECOND algorithm and BiFPN work together to refine the fused features, resulting in improved multi-modal perception performance. This multi-stage fusion process ensures that both the spatial and semantic aspects of the multimodal data are effectively integrated, leading to significant improvements in the accuracy and efficiency of the perception system.

### Detection head and loss function

Building on the detection head design in TransFusion-L, we propose a similar yet modified detection head and loss function, capable of handling both LiDAR and image data. The following outlines the design approach.

#### Detection head.

We utilize an adaptive heatmap that dynamically adjusts its resolution based on the size of the objects and the complexity of the scene. This feature improves object localization in dense environments. Unlike class-specific heatmaps, we employ a multimodal fusion heatmap that combines information from both LiDAR and image data to enhance the robustness of center predictions. Similar to TransFusion-L, a regression head predicts the object’s size, velocity, and rotation angle. Additionally, we incorporate semantic context, such as road conditions and object motion trends, to improve prediction accuracy. A cross-modal attention mechanism is implemented to allow interaction between LiDAR and image features. This ensures that object predictions consider the complementary advantages of both modalities. Sparse object queries and self-attention mechanisms are used to iteratively refine object queries, rather than simply aggregating features. This process enhances the accuracy of object predictions by refining each query through attention.

#### Loss function.

We design a loss function similar to that of TransFusion-L, addressing issues such as class imbalance, bounding box localization, and center heatmap loss.

Total Loss Function:

$$L_{\text{fusion}}=L_{\text{bbox}}+L_{\text{cls}}+L_{\text{heatmap}}+L_{\text{semantic}}$$ (11)

We use the Smooth L1 loss (Huber loss) instead of the L1 loss, providing more stability when handling outliers and smoother training progress. Bounding Box Regression Loss:

$$L_{\text{bbox}}=\sum_{i}\text{SmoothL1}(B_{i}-{\hat{B}}_{i})$$ (12)

where *B*_*i*_ is the ground truth bounding box and ${\hat{B}}_{i}$ is the predicted bounding box.

To address class imbalance, we replace focal loss with balanced cross-entropy loss:

$$L_{\text{cls}}=-\frac{1}{N}\sum_{i}w_{i}\cdot \left(y_{i}\log({\hat{p}}_{i})+(1-y_{i})\log(1-{\hat{p}}_{i})\right)$$ (13)

where *w*_*i*_ is the class weight factor to balance class distribution.

We use a Gaussian heatmap loss with IoU weighting to impose a stronger penalty for localization errors, especially for highly overlapping objects. Heatmap Loss (Gaussian Heatmap + IoU-weighted):

$$L_{\text{heatmap}}=\sum_{i}({\hat{h}}_{i}-h_{i}{)}^{2}\cdot \text{IoU}(B_{i},{\hat{B}}_{i})$$ (14)

where *h*_*i*_ is the ground truth heatmap, and ${\hat{h}}_{i}$ is the predicted heatmap.

Given the multimodal nature of the data, semantic loss ensures that detected objects align with predefined semantic categories (e.g., vehicles, pedestrians). This loss function serves as an additional term in the total loss. Semantic Loss:

$$L_{\text{semantic}}=-\sum_{i}\log(p_{i})$$ (15)

where *p*_*i*_ is the predicted probability of the object’s semantic category.

## Experiments

### Dataset

nuScenes dataset is a comprehensive, large-scale dataset developed by Motional for autonomous driving research [68]. It features a diverse collection of 1,000 driving scenes from two cities, Boston and Singapore, renowned for their challenging traffic conditions. Each scene is 20 seconds long and showcases various driving maneuvers, traffic situations, and unexpected events, reflecting the complexities of urban driving. The dataset includes rich multi-modal sensor data from a full sensor suite, consisting of 6 cameras, 1 LiDAR, 5 radars, GPS, and IMU.

nuScenes offers 23 object categories annotated with accurate 3D bounding boxes at 2Hz, making it ideal for object detection and tracking tasks. In addition, object attributes such as visibility, activity, and pose are annotated, enhancing its utility for advanced perception tasks. The dataset contains approximately 1.4 million images from cameras, 390,000 LIDAR sweeps, 1.4 million RADAR sweeps, and 1.4 million object bounding boxes in 40,000 keyframes.

### Evaluation criteria

The LGMMfusion detection task involves detecting 10 object categories, with each object annotated with 3D bounding boxes, attributes, and velocities. To assess the accuracy of the detection, matching is performed by calculating the distance *d* between the 2D center of the predicted 3D bounding box on the ground plane and the actual ground truth. Predictions with recall or precision lower than 10 % are filtered out to ensure only relevant matches are considered. The Average Precision (AP) is then calculated as the normalized area under the precision-recall curve.The overall mean Average Precision (mAP) is defined as:

$$\text{mAP}=\frac{1}{\left|C\right|}\sum_{c\in C}\left(\frac{1}{\left|D\right|}\sum_{d\in D}{\text{AP}}_{c,d}\right)$$ (16)

where: *C* denotes the set of predicted classes, *D* represents the set of distance thresholds between the predicted and true centers on the ground plane, ${\text{AP}}_{c,d}$ is the Average Precision for a class *c* and distance threshold *d*. For our evaluation, we adopt the following distance thresholds D={0.5,1.0,2.0,4.0}.

Additionally, five key metrics for True Positives (TP) are computed during the matching process: Average Translation Error (ATE): Measures the translation accuracy of the detected object. Average Scale Error (ASE): Evaluates the accuracy of the object scale prediction. Average Orientation Error (AOE): Assesses the rotational accuracy of the detected object. Average Velocity Error (AVE): Quantifies the error in velocity prediction. Average Attribute Error (AAE): Measures how accurately the object attributes, such as visibility or activity, are predicted.Each of these metrics is calculated based on a 2-meter center distance threshold, ensuring robust performance metrics for localization and object behavior prediction.To summarize the detection performance, we compute the mean True Positive rate (mTP) as:

$$\text{mTP}=\frac{1}{\left|C\right|}\sum_{c\in C}TP_{c}$$ (17)

where *TP*_*c*_ represents the number of True Positives for class *c*. Finally, the nuScenes detection score Detection Score (NDS), which serves as the integrated metric combining both detection performance and quality, is calculated as follows:

$$\text{NDS}=\frac{1}{10}\left(5\cdot \text{mAP}+\sum_{mTP\in TP}(1-\min(1,\text{mTP}))\right)$$ (18)

This score reflects the overall effectiveness of the detection system by balancing both the precision of the object detection (via mAP) and the quality of the object localization (via mTP). By leveraging these evaluation metrics, LGMMfusion provides a comprehensive and reliable performance measure for autonomous driving detection tasks.

### Experimental settings

**Model Setup:** The LGMMfusion network is built upon the open-source 3D object detection framework mmdetection3D. Our framework is implemented using PyTorch, which is optimized for training on GPUs. For image feature extraction, we use a pre-trained Swin-Tiny model, which has been pre-trained on the ImageNet dataset. To optimize training efficiency and reduce GPU memory usage, we freeze the weights of the pre-trained model during the entire training process. For LiDAR feature extraction, the point cloud is voxelized, and sparse convolutions are used to extract the features from the voxelized data.

In the process of generating the BEV image from the LiDAR data, we utilize 6 layers of MHMSSA and MHACA. Additionally, we account for the effects of data augmentation on the transformation matrix $M_{\text{lidar2img}}$. This transformation matrix is updated based on the augmentation matrices $M_{\text{img aug}}$ and $M_{\text{lidar aug}}$. The input image size is set to 256x704 pixels, and the voxel size is defined as [0.075m,0.075m,0.2m]. The BEV feature map resolution is set to 256×704 based on the voxel grid, with each voxel representing 0.075 meters in the X and Y dimensions and 0.2 meters in the Z dimension. During image BEV feature generation, we adopt deformable attention with 8 sampling points per reference and 4 spatial scales, which aligns with the structure of our multi-head adaptive cross-attention mechanism.

**Training Details:** The LGMMfusion network is trained using PyTorch 2.1.0 with CUDA 11.8 and cuDNN 8.6. For end-to-end training, we leverage a distributed setup with 8 NVIDIA A100 GPUs. In the first phase, we train the LiDAR branch of the network for 25 epochs using the AdamW optimizer with a learning rate of 0.00005. In the second phase, the image branch is introduced, and training continues for another 12 epochs, using the same AdamW optimizer with a learning rate of 0.00005. During inference, we do not use test-time augmentation or multi-model ensembling to ensure consistent results across different experiments.

### Experimental results and analysis

We conducted extensive experiments to evaluate the performance of our proposed method, LGMMfusion, and compared it with several state-of-the-art approaches. These include camera-based methods FCOS3D [70] , PGD [71] and BEVFormer [49], LiDAR-based methods such as PointPillars [69], SECOND [43], and CenterPoint [72], as well as LiDAR-camera fusion models like CFF [74], UVTR [20], mmFusion [78], and FUTR3D [64]. The results on the nuScenes validation set are presented in Table 1.

**Table 1:** Results on nuScenes val set. The modalities are Camera (C), LiDAR (L).

Metric	Cite	Modality	mAP ↑	NDS ↑	mATE ↓	mASE ↓	mAOE ↓	mAVE ↓	mAAE ↓
FCOS3D	[70]	C	0.343	0.415	0.725	0.263	0.422	1.292	0.153
PGD	[71]	C	0.369	0.428	0.683	0.260	0.439	1.268	0.185
BEVFormer	[49]	C	0.416	0.517	0.672	0.273	0.371	0.392	0.199
PointPillars	[69]	L	0.516	0.621	-	-	-	-	-
SECOND	[43]	L	0.528	0.629	-	-	-	-	-
CenterPoint	[72]	L	0.532	0.649	-	-	-	-	-
CFF	[74]	L+C	0.651	0.689	-	-	-	-	-
UVTR	[20]	L+C	0.651	0.698	0.331	0.259	**0.269**	**0.221**	0.176
mmFusion	[78]	L+C	0.654	0.697	-	-	-	-	-
FUTR3D	[64]	L+C	0.666	0.683	0.274	0.258	0.315	0.255	0.178
**LGMMfusion**	Ours	L+C	**0.673**	**0.711**	**0.266**	**0.252**	0.311	0.248	**0.173**

The experimental results demonstrate that LGMMfusion achieves significant performance improvements over both pure camera-based and multi-modal fusion methods. Specifically, compared to pure camera-based approaches, LGMMfusion outperforms FCOS3D and PGD by a large margin. FCOS3D and PGD exhibit lower detection accuracy, with mAP scores of 0.343 and 0.369, respectively, significantly trailing behind LGMMfusion’s 0.673. This discrepancy highlights the inherent limitations of monocular 3D object detection, particularly in estimating accurate depth and spatial localization. Additionally, their lower NDS values (0.415 and 0.428) further confirm the challenge of achieving robust detection using camera-only methods, reinforcing the necessity of integrating LiDAR data to improve overall perception accuracy. Furthermore, compared to BEVFormer, a more advanced camera-based method, LGMMfusion still demonstrates substantial advantages. Our approach achieves a relative improvement of 25.7% in mAP and 19. 4% in NDS, together with notable reductions in mATE and mASE. These improvements emphasize the effectiveness of leveraging both LiDAR and camera data for enhanced detection performance. The ability of LGMMfusion to incorporate precise geometric depth information from LiDAR while preserving rich semantic cues from camera data enables it to achieve superior accuracy, particularly in challenging scenarios with occlusions and distant objects.

Comparing LGMMfusion with LiDAR-based methods like PointPillars, SECOND, and CenterPoint, our model demonstrates substantial improvements in both mAP and NDS. LGMMfusion achieves an mAP improvement of 15.7%, 14.5%, and 14.1% over PointPillars, SECOND, and CenterPoint, respectively. In terms of NDS, our model surpasses these methods by 9.0%, 8.2%, and 6.2%, respectively, demonstrating the benefits of multi-modal fusion over LiDAR-only approaches.

When compared with existing LiDAR-camera fusion models such as CFF, UVTR, mmFusion, and FUTR3D, LGMMfusion also achieves state-of-the-art performance. Our method surpasses CFF and UVTR by 2.2% in mAP, mmFusion by 1.9%, and FUTR3D by 0.7%. Furthermore, in terms of NDS, LGMMfusion achieves an improvement of 2.2% over CFF and UVTR, 1.4% over mmFusion, and 2.8% over FUTR3D.

One notable observation is that despite being purely camera-based, BEVFormer demonstrates a competitive performance compared to other vision-only methods, with an mAP of 0.416 and an NDS of 0.517. However, its performance still lags significantly behind multi-modal fusion methods, particularly in localization accuracy, as indicated by its higher mATE value (0.672). This further supports the argument that while advanced camera-based approaches can achieve moderate accuracy, the absence of depth-aware LiDAR information remains a critical bottleneck.

In terms of error metrics, LGMMfusion reduces mATE, mASE, and mAAE compared to other methods, demonstrating improved localization accuracy and robustness. In particular, our method achieves a 40.6% reduction in mATE compared to BEVFormer, indicating significantly enhanced depth estimation and object localization.

Overall, the experimental results on the nuScenes dataset confirm that LGMMfusion effectively combines LiDAR and camera data, resulting in a substantial performance boost in 3D object detection. Our method consistently outperforms both LiDAR-only and camera-only approaches across multiple evaluation metrics, reinforcing the advantages of multi-modal fusion.

To further emphasize the timeliness of our approach, we compare LGMMfusion against several recently proposed multimodal 3D detection frameworks, including FUTR3D [64], UVTR [20], and mmFusion [78]. As shown in Table 2, LGMMfusion outperforms these methods in both mAP and NDS. In particular, it improves small object detection categories such as pedestrians and traffic cones by 2.5–5.0%, highlighting the effectiveness of our LiDAR-guided BEV feature alignment. Unlike FUTR3D, which fuses image features at the proposal level, our method performs early spatial alignment at the BEV level, enabling more coherent feature representation for dense urban scenes. These comparisons confirm that LGMMfusion remains competitive with and complementary to the latest state-of-the-art methods in multimodal perception.

**Table 2:** Results on the nuScenes validation set. The results are compared across different methods using LiDAR (L) and Camera (C) modalities(Mod). The performance is evaluated both overall (mAP, NDS) and at the per-class level [75]. The classes include Car, Truck(Tru), Construction Vehicle (C.V.), Bus, Trailer(Tra), Barrier(Bar), Motorcycle (Motor), Bike, Pedestrian (Ped.), and Traffic Cone (T.C.).   Small object categories include pedestrian, traffic cone, and bicycle, which show the most notable mAP improvements.

Metric	Mod	mAP ↑	NDS ↑	Car	Tru	C.V.	Bus	Tra	Bar	Motor	Bike	Ped.	T.C.
CenterPoint [76]	L	53.2	64.9	85.7	58.9	17.1	71.4	37.0	68.8	59.1	43.3	85.4	69.7
CFF [77]	L+C	65.1	68.9	85.8	59.1	22.5	73.4	43.2	68.7	73.2	62.4	86.4	70.6
mmFUSION [78]	L+C	65.4	69.7	86.0	61.0	28.5	72.9	40.6	67.1	74.0	65.2	85.2	72.5
FUTR3D [64]	L+C	66.6	68.3	86.2	61.8	25.9	72.0	42.1	64.4	73.6	63.2	82.9	70.4
LGMMfusion(Our)	L+C	67.3	71.1	89.0	60.7	29.7	76.1	42.2	71.4	73.5	59.2	88.4	77.5

In addition to detection performance, we also report the computational complexity of LGMMfusion. The model requires approximately 125 GFLOPs per frame and achieves an inference speed of 12.3 FPS on an NVIDIA A100 GPU. In comparison, CenterPoint consumes 92 GFLOPs and runs at 19.4 FPS under the same conditions. While LGMMfusion introduces higher computational cost due to image BEV generation and attention mechanisms, it achieves significantly better detection performance, especially on small and occluded objects. This trade-off is acceptable for high-accuracy perception scenarios such as autonomous driving.

**Fig 5:**
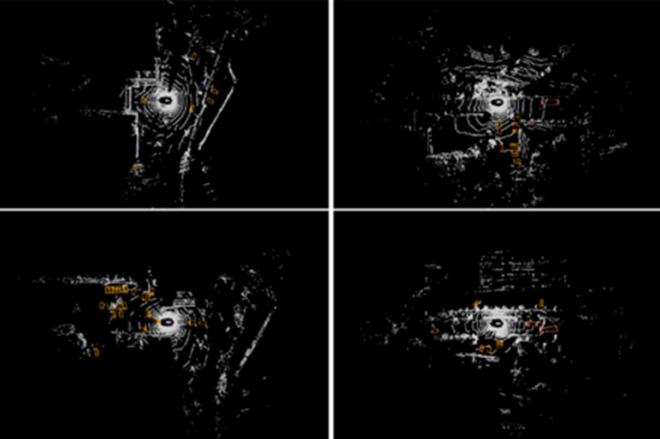
This figure shows an example of 3D annotation, each row represents a scene. The first row is a tunnel during the day, and the second row is a tunnel at night.

Fig 5 visualizes the detection results in the BEV framework. Our approach effectively identifies objects even in low-density point cloud regions. This improvement is primarily due to the synergistic integration of LiDAR and image features, where high-resolution semantic details from the image modality compensate for sparse LiDAR information, particularly for distant and small-scale objects.

Table 2 presents the 3D detection results on the nuScenes validation set, where we compare the performance of LGMMfusion with several existing methods, including CenterPoint, CFF, mmFUSION, and FUTR3D. The evaluation metrics include mean Average Precision (mAP) and NuScenes Detection Score (NDS), as well as per-class performance for several object categories.

Overall, LGMMfusion achieves competitive performance with an mAP of 67.3% and NDS of 71.1%, showing noticeable improvements over CenterPoint and FUTR3D in several categories. For instance, the Traffic Cone (T.C.) class shows a significant improvement of 5% over mmFUSION, while the Bus category shows a modest gain of 4.1% compared to FUTR3D. These results indicate that our method performs well in detecting smaller and more challenging objects.In terms of class-specific performance, LGMMfusion also shows reasonable gains in categories like Pedestrian (Ped.) and Motorcycle (Motor.), with mAP improvements of approximately 3.0% and14.4% over CenterPoint, respectively. These gains suggest that LGMMfusion benefits from its fusion of LiDAR and camera modalities, particularly in handling small-sized objects where traditional methods may struggle.

In summary, LGMMfusion demonstrates solid improvements, particularly in detecting smaller objects like Pedestrian and Traffic Cone, but the enhancements are more moderate in larger object categories. The results validate the effectiveness of our multi-modal fusion approach in enhancing the detection of various object scales without drastic performance trade-offs.

### Ablation study

In this section, we present the results of our ablation study on the nuScenes validation set, as shown in Table Table 3. The purpose of this experiment was to investigate the impact of different components of the LGMMfusion model. We evaluated four model variants: a baseline using LiDAR data only, and three additional configurations incorporating camera data and variations in the Image BEV processing.

**Table 3:** Extended ablation study results on the nuScenes validation set. This table compares the performance of LGMMfusion variants, including the impact of Image BEV (I-BEV), BEV Query (BEV-Q), attention structure modules (MHMS-SA and MHA-CA), and attention parameters (number of heads *H* and scales *S*). I-BEV variations involve the use of BatchNorm (BN) and ReLU activations.

Variant	mAP ↑	NDS ↑	mATE ↓	mASE ↓	mAOE ↓	mAVE ↓	mAAE ↓
L only (baseline)	63.4	68.8	0.2821	0.2512	0.3162	0.2547	0.1893
L+C, w/o BEV-Q, I-BEV (BN)	65.1	69.2	0.2831	0.2571	0.2953	0.2819	0.1909
L+C, w/ BEV-Q, I-BEV (BN)	66.3	70.1	0.2812	0.2568	0.3370	0.2869	0.1843
L+C, w/ BEV-Q, I-BEV (BN+ReLU)	**67.3**	**71.1**	0.2612	0.2514	0.3112	0.2491	0.1729
w/o MHMS-SA	66.2	70.0	0.2685	0.2543	0.3181	0.2590	0.1814
w/o MHA-CA	65.9	69.7	0.2704	0.2536	0.3245	0.2615	0.1831
*H* = 4, *S* = 2	66.5	70.3	0.2648	0.2541	0.3147	0.2558	0.1778
*H* = 8, *S* = 4 (default)	67.3	71.1	0.2612	0.2514	0.3112	0.2491	0.1729
*H* = 12, *S* = 6	67.4	70.9	0.2630	0.2527	0.3091	0.2504	0.1732

As the baseline, the LiDAR-only model achieved an mAP of 63.4 and an NDS of 68.8. This configuration serves as a reference point for assessing the performance improvements introduced by incorporating additional data modalities and processing strategies. When the camera modality was introduced along with BatchNorm (BN) processing, the model exhibited a notable improvement, with mAP increasing to 65.1 and NDS reaching 69.2. This demonstrates that leveraging camera information, even with basic normalization techniques, enhances detection accuracy and object localization by enriching semantic context and structural details.Further performance gains were observed when the BEV Query (BEV-Q) mechanism was introduced, leading to an mAP increase to 66.3 and an NDS improvement to 70.1. The 1.2% rise in mAP over the previous configuration indicates that BEV Query effectively refines the representation of fused LiDAR and camera features, enabling more accurate object detection in 3D space. This suggests that dynamically querying BEV features facilitates better spatial feature alignment, improving object representation across different sensor modalities. The final configuration, which incorporates ReLU activation into Image BEV processing, yielded the highest performance, with mAP increasing to 67.3 and NDS reaching 71.1. This improvement highlights the crucial role of non-linearity in refining feature transformations. ReLU enhances the expressiveness of extracted image features, allowing for more robust feature interactions between LiDAR and camera data. Furthermore, the noticeable reductions in mATE and mAOE indicate improved localization precision and orientation estimation, confirming the positive impact of this enhancement.

In summary, each modification introduced in this study contributed to progressive improvements in detection performance. The integration of camera data with LiDAR provided a solid foundation for multimodal fusion, while the addition of BEV Query and ReLU further refined feature alignment and representation. These results emphasize the effectiveness of our fusion strategy in LGMMfusion and demonstrate the critical role of both advanced processing techniques and multimodal data integration in optimizing 3D object detection.

To further analyze the contribution of individual modules, we performed additional ablation experiments by disabling the MHMS-SA and MHA-CA blocks separately. Removing the MHMS-SA module reduced mAP from 67.3% to 66.2%, while disabling the MHA-CA module resulted in an mAP of 65.9%. These results confirm that both components play critical roles in enhancing feature representation and cross-modal alignment. We also conducted a sensitivity analysis on the number of attention heads (*H*) and the number of spatial scales (*S*) used in the deformable attention mechanisms. The model achieves optimal performance when *H* = 8 and *S* = 4. Increasing these values beyond this point yields negligible improvement or mild overfitting, whereas reducing them leads to degraded detection accuracy. Detailed results are included in Table 3.

## Discussion

In this section, we analyze the experimental results of LGMMfusion, focusing on its performance in 3D object detection, the impact of image BEV generation, and practical considerations such as computational cost and applicability. Additionally, we discuss the limitations of our approach and outline potential directions for future research.

The experimental results demonstrate that LGMMfusion achieves high detection accuracy in complex urban environments, effectively handling occlusions, small objects, and challenging object distributions. Compared to BEVFormer, a camera-based method, LGMMfusion exhibits substantial improvements in mAP and NDS, underscoring the importance of LiDAR for depth-aware perception. Furthermore, our method outperforms other LiDAR-camera fusion approaches, such as FUTR3D and mmFusion, highlighting the effectiveness of our multi-modal feature alignment strategy. In addition to quantitative improvements, LGMMfusion also demonstrates strong qualitative performance. As shown in Fig 5, our model accurately detects continuously distributed obstacles, densely packed pedestrians, and indistinct objects such as blurred bicycles. The regions highlighted in red indicate areas with severe occlusion or small-sized objects, which are particularly challenging for conventional detection models. To better illustrate our method’s effectiveness, we have magnified and annotated these regions. This capability significantly reduces missed detections, thereby improving NDS and reinforcing the robustness of our approach in complex environments. Furthermore, ablation studies confirm that image BEV generation enhances detection accuracy, with notable improvements in mAP and NDS when BEV Query and ReLU are incorporated.

To further quantify the model’s effectiveness on small object categories, we analyzed the detection performance on pedestrians and traffic cones—two classes with small physical sizes and sparse LiDAR reflections. As shown in Table 2, LGMMfusion improves the AP for pedestrians by 3.0% and traffic cones by 5.0% compared to mmFusion. These gains are particularly meaningful in autonomous driving, where small objects often correspond to vulnerable road users. Additionally, we conducted a subset-based evaluation under different environmental conditions. On nuScenes validation scenes labeled as ’night’ or ’rain’, LGMMfusion maintains a consistent improvement in mAP (1.7%–2.1%) over mmFusion and CenterPoint. These results demonstrate the model’s robustness under adverse visibility conditions and further support its practical applicability.

From a safety perspective, LGMMfusion demonstrates measurable performance gains on categories that are directly associated with accident risk in autonomous driving scenarios. For example, our method achieves a 3.0% improvement in mAP for pedestrians and a 5.0% gain for traffic cones compared to mmFusion. These object types are critical for ensuring safe navigation, especially in urban environments. Although our study does not directly quantify accident reduction rates, such improvements in the detection of vulnerable road users are strongly correlated with reduced perception failures and enhanced collision avoidance. Therefore, the observed improvements support LGMMfusion’s potential for increasing real-world safety in autonomous systems.

While LGMMfusion delivers superior performance, it introduces additional computational overhead due to BEV feature extraction and fusion. This trade-off between accuracy and efficiency is particularly relevant for real-time applications such as autonomous driving, where both detection precision and inference speed are crucial. Additionally, our method relies on accurate sensor calibration, which may limit its robustness in environments with poor synchronization or adverse weather conditions. Future research could focus on optimizing computational efficiency to enable real-time deployment in resource-constrained settings. Furthermore, extending LGMMfusion to tasks such as segmentation and tracking could further enhance its applicability in 3D perception. Investigating adaptive sensor fusion techniques to mitigate calibration dependencies and exploring lightweight architectures to reduce computational complexity are promising directions for future work.

Despite its advantages, LGMMfusion inherits certain robustness limitations common to multi-sensor systems. Notably, the framework assumes accurate extrinsic calibration between LiDAR and camera sensors, and performance may degrade in the presence of spatial misalignment, such as in dynamic mechanical setups or under vibration. Furthermore, its reliance on clean multi-modal inputs makes it susceptible to degraded sensor signals in adverse weather (e.g., rain, fog) or occlusion-heavy scenes. To address these issues, future extensions of LGMMfusion may incorporate online self-calibration modules that dynamically adjust fusion parameters during operation. Additionally, confidence-aware or attention-gated fusion strategies could help the model adaptively weight reliable sensor inputs. Incorporating multi-frame temporal fusion and lightweight redundancy-aware modules may further enhance robustness and temporal stability in real-world deployment.

## Conclusion

In this paper, we propose a novel 3D object detection method, LGMMfusion, which integrates a LiDAR depth-guided image BEV generation module and effectively fuses image BEV with LiDAR BEV. Experimental results on the nuScenes dataset demonstrate that our method achieves superior detection performance, leveraging multi-modal data to surpass many traditional detection approaches. In particular, LGMMfusion significantly enhances the detection of small and distant objects, as well as heavily occluded targets, by exploiting the complementary strengths of LiDAR and camera modalities. Despite these advantages, several challenges remain. First, while LGMMfusion achieves high accuracy, it introduces additional computational overhead, which may hinder real-time deployment in resource-constrained environments. Second, our approach relies on precise sensor calibration; misalignment between LiDAR and camera data can degrade fusion effectiveness. Third, our model has primarily been evaluated in urban driving scenarios, and its generalization to more diverse environments, such as off-road conditions or adverse weather scenarios, requires further investigation. Unlike prior intermediate fusion approaches such as BEVFusion and FUTR3D, LGMMfusion uniquely incorporates LiDAR-derived geometric priors to guide image BEV generation and cross-modal feature alignment. This geometry-aware fusion design enhances the spatial consistency and robustness of multimodal 3D perception, particularly in complex urban environments. Future work will focus on addressing these limitations by optimizing the computational efficiency of LGMMfusion to enable real-time inference, developing adaptive sensor fusion techniques to mitigate calibration dependency, and extending the framework to handle more diverse and challenging environments. Furthermore, exploring lightweight architectures and self-supervised learning strategies could enhance the model’s applicability in large-scale autonomous systems.
